# Enrichment of human nasopharyngeal bacteriome with bacteria from dust after short-term exposure to indoor environment: a pilot study

**DOI:** 10.1186/s12866-023-02951-5

**Published:** 2023-07-31

**Authors:** Eva Konecna, Petra Videnska, Lucie Buresova, Milan Urik, Sona Smetanova, Stanislav Smatana, Roman Prokes, Barbara Lanickova, Eva Budinska, Jana Klanova, Petra Borilova Linhartova

**Affiliations:** 1 RECETOX, Faculty of Science, Masaryk University, Kotlarska 2, Brno, Czech Republic; 2 Department of Pediatric Otorhinolaryngology, University Hospital Brno, Černopolní 9, 613 00 Brno, Czech Republic; 3 Department of Pediatric Otorhinolaryngology, Faculty of Medicine, Masaryk University, Kamenice 5, Brno, Czech Republic; 4 Global Change Research Institute of the Czech Academy of Sciences, Bělidla 986/4a, Brno, Czech Republic; 5 Department of Gynaecology and Obstetrics, University Hospital Brno, Obilni Trh 526/11, 602 00 Brno, Czech Republic

**Keywords:** Bacteriome, Dust, Nasopharynx, Household, Hospital, Sequencing, 16S rRNA, Exposure, Indoor environment

## Abstract

**Background:**

Indoor dust particles are an everyday source of human exposure to microorganisms and their inhalation may directly affect the microbiota of the respiratory tract. We aimed to characterize the changes in human nasopharyngeal bacteriome after short-term exposure to indoor (workplace) environments.

**Methods:**

In this pilot study, nasopharyngeal swabs were taken from 22 participants in the morning and after 8 h of their presence at the workplace. At the same time points, indoor dust samples were collected from the participants’ households (16 from flats and 6 from houses) and workplaces (8 from a maternity hospital – NEO, 6 from a pediatric hospital – ENT, and 8 from a research center – RCX). 16S rRNA sequencing analysis was performed on these human and environmental matrices.

**Results:**

*Staphylococcus* and *Corynebacterium* were the most abundant genera in both indoor dust and nasopharyngeal samples*.* The analysis indicated lower bacterial diversity in indoor dust samples from flats compared to houses, NEO, ENT, and RCX (*p* < 0.05). Participants working in the NEO had the highest nasopharyngeal bacterial diversity of all groups (*p* < 0.05). After 8 h of exposure to the workplace environment, enrichment of the nasopharynx with several new bacterial genera present in the indoor dust was observed in 76% of study participants; however, no significant changes were observed at the level of the nasopharyngeal bacterial diversity (*p* > 0.05, Shannon index). These “enriching” bacterial genera overlapped between the hospital workplaces – NEO and ENT but differed from those in the research center – RCX.

**Conclusions:**

The results suggest that although the composition of nasopharyngeal bacteriome is relatively stable during the day. Short-term exposure to the indoor environment can result in the enrichment of the nasopharynx with bacterial DNA from indoor dust; the bacterial composition, however, varies by the indoor workplace environment.

**Supplementary Information:**

The online version contains supplementary material available at 10.1186/s12866-023-02951-5.

## Background

Nowadays, people spend most of their time indoors in constant contact with various microorganisms interacting, positively or negatively, with the human organism. Dust is an important reservoir of microorganisms in the building interiors. The indoor dust bacteriome may be unique for each building as its formation is influenced by multiple factors. In general, the composition and diversity of microorganisms associated with indoor dust are given by a dynamic interaction between the building itself (including ventilation methods, room temperature, air humidity, and materials used in the building) [1–3], the outside environment (geographic location, climate, air pollution) [4–6], and the building’s occupants (humans, animals) [7–9].

Numerous studies have demonstrated that humans serve as the main reservoir of indoor microorganisms as they emit significant amounts of bacteria that originate mainly from their skin and gastrointestinal tract. Even everyday human activities such as talking, walking, cleaning, vacuuming, or using toilets and showers contribute to the spreading of microorganisms [10, 11]. On the other hand, the high content of bacteria specific to the skin, nostrils, and hair of humans found in indoor floor dust indicates that floors are an important reservoir of human-associated bacteria [9]. *Staphylococcus*, *Corynebacterium*, and *Streptococcus* are the most prevalent bacterial genera in indoor dust [12]. Previous research [9] indicates that the number of occupants significantly impacts the indoor dust bacteriome. Commercially used buildings such as offices, hospitals, or shopping centers have a high number of visitors per day and, thus, a greater diversity of the dust bacteriome, which is constantly changing due to new arrivals [13]. Furthermore, dust in hospitals can contain a higher concentration of pathogenic and antimicrobial-resistant microorganisms due to the frequent use of disinfectants and antimicrobials [14, 15]. In contrast, the turnover of people in households is lower than in public buildings and, therefore, the indoor dust bacteriome in households is not subject to rapid changes [16].

In recent years, the dust microbiome has received a lot of attention because it plays an essential role in the development of various diseases, such as chronic respiratory diseases [17]. Due to public health concerns, many studies have investigated indoor microbiome in different environments such as households [17–21], offices [13, 22, 23], schools and childcare centers [24–26], hospitals [27–30], farms [31, 32], airplanes [33–35], and even the International Space Station [36]. The indoor dust microbiomes from households and schools were analyzed by Hanson et al. [12], who found that the bacterial community in the dust from the classroom floor differed from typical house dust, which contains more gram-positive and human-associated bacteria [37]. Other studies compared the microbiome inside and outside buildings [38, 39]. Gupta et al. explored the influence of environmental factors on bed dust bacteriome and mycobiome and their correlations with airway bacteriome in early life [40]. However, there is a lack of data providing information on the effects of short-term human exposure to the indoor dust in buildings where people spend most of their time (i.e. home, work) on the composition of their upper respiratory tract bacteriome.

The upper respiratory tract consists of the nose and nasal cavity, paranasal cavities, nasopharynx, and oropharynx; these structures differ anatomically and form different microenvironments with different microbial compositions [41, 42]. It is the gateway for microorganisms, which can be inhaled with air and dust particles from the surrounding environment. Many factors can influence the structure of the human respiratory bacteriome, including acute and chronic respiratory diseases [43–50], age [51], smoking [52–55], and air pollution [42, 56–58]. During the COVID-19 pandemic, the nasopharyngeal microbiome came to the forefront of research, and the number of studies dealing with the microbiome in this location has increased rapidly [59]. De Boeck et al. previously suggested that the nasopharyngeal bacteriome of healthy adults can be classified into four profiles according to the most dominant bacterial genera: 1) *Moraxella* type, 2) *Fusobacterium* type, 3) *Streptococcus* type, 4) Mixed *Corynebacterium*, *Staphylococcus*, and *Dolosigranulum* type [60].

We aimed to provide pilot data on the human nasopharyngeal bacteriome after short-term exposure (8 h) to indoor environments at participants’ workplaces because we hypothesized that exposure to microbial communities in the surrounding environment might affect the bacteriome of the upper respiratory tract. Our main goal was to investigate whether the indoor dust bacteriome affects the nasopharyngeal bacteriome. The specific aims were i) to get insight into the variability of the dust bacteriome in households and three different workplaces (two hospitals and one research center) and ii) to compare nasopharyngeal bacteriomes in the morning and after 8 h at the workplace, i.e., to describe changes between these two timepoints and differences between the groups of participants. The unique design of our study provides a first peek into the complex information on the effects of the indoor dust bacteriome on the human nasopharyngeal bacteriome composition.

## Results

A total of 22 adult volunteers working at 3 different workplaces were recruited – 8 participants from the maternity hospital – Department of Gynaecology and Obstetrics (Neonatology, NEO), University Hospital Brno, 6 from the pediatric hospital – Department of Pediatric Otorhinolaryngology (Ear, Nose, and Throat, ENT), University Hospital Brno, and 8 from the research center – RECETOX (RCX) at Masaryk University, Brno. A total of 88 samples were sequenced, of which 44 were dust samples and 44 nasopharyngeal swabs.

After quality filtering and chimeras removal, we obtained 4,647,653 reads (median = 43,772 reads per sample; interquartile range, IQR = 48,525), of which 2,382,614 reads (median = 49,192 reads per sample; IQR = 21,679) originated from the dust samples (*n* = 44) and 2,265,039 reads (median = 23,470 reads per sample; IQR = 66,050) from the nasopharyngeal swabs (*n* = 43); one nasopharyngeal swab sample collected in the afternoon from a participant working at ENT (Np.ENT.22.Aft, E6) was excluded from further analyses due to an insufficient amount of isolated DNA.

Most phyla (90.0% in the dust samples and 94.9% in the nasopharyngeal samples) were present at low abundances (median relative abundance of < 5%). In the dust samples, *Actinobacteria* were the most abundant phyla, comprising in median 36.8% of filtered genera, followed by *Firmicutes* with a median of 26.9% and Proteobacteria with a median of 25.4%. These three major phyla comprised in median 94.7% of filtered genera. In the nasopharyngeal samples, *Firmicutes* were the most abundant phyla, comprising in median 40.4% of filtered genera, followed by *Actinobacteria*, comprising in median 24.1% of filtered genera. Together, these two most common phyla comprised in median 87.4% of filtered genera. The median of unassigned reads at genus level was 27.7% of the reads in dust samples and 3% of the reads in nasopharyngeal samples.

Dust samples and nasopharyngeal swabs were evaluated separately. The diagram of PERMANOVA testing and its result (Additional file 1) shows all variables tested.

### Indoor dust bacteriome

Overall, we found 1,444 genera representing 40 phyla in the dust microbiome (Additional file 2). *Corynebacterium 1* (median 7.9%), and *Staphylococcus* (median 6.8%) were the most abundant genera in dust samples (Additional file 3).

**Fig. 1:**
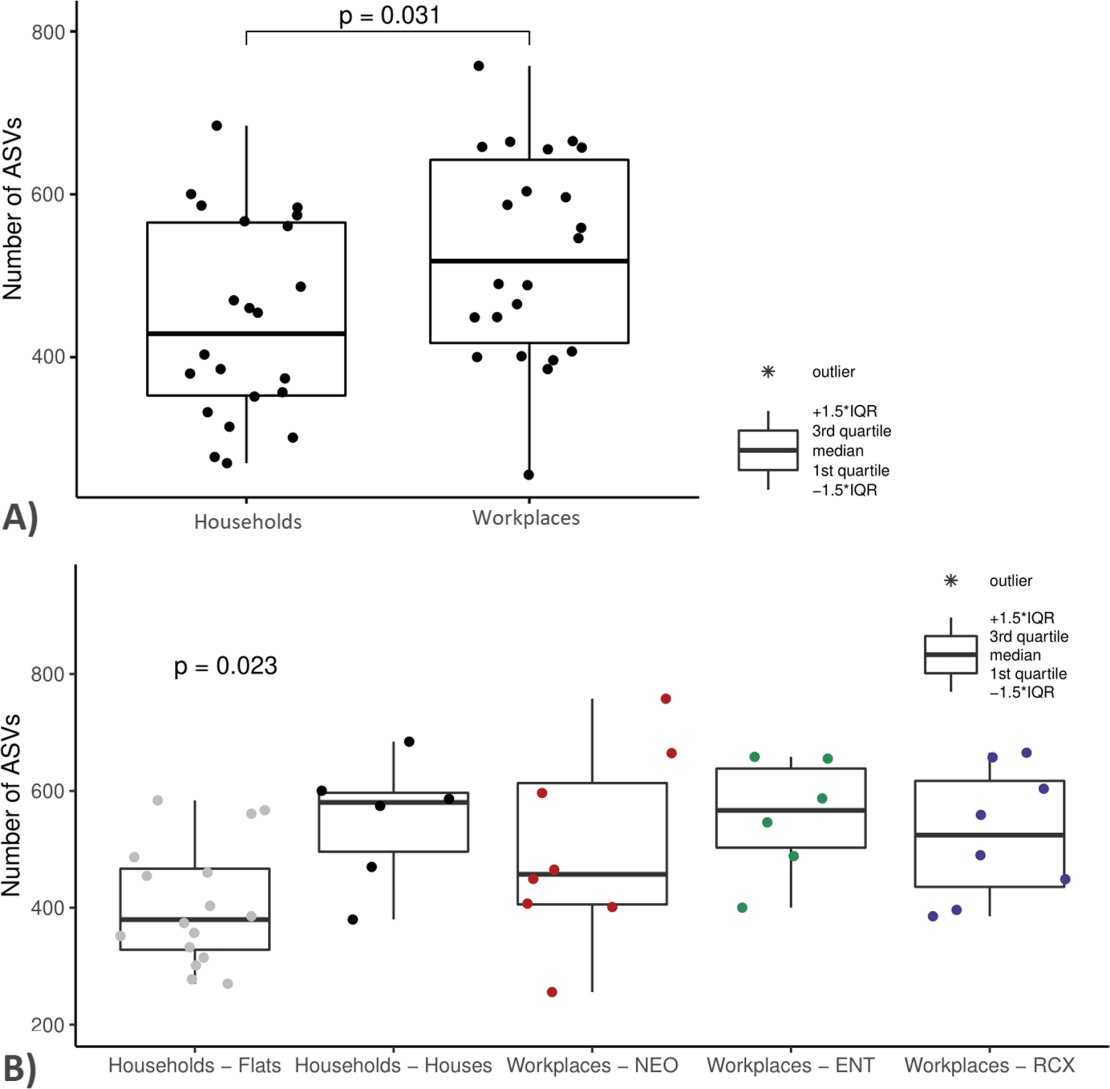
Comparison of bacterial diversity (the number of ASVs) in dust samples from households and workplaces. **A** Comparison of dust samples from households (*n* = 22) and workplaces (*n* = 22) tested by Mann–Whitney test (*p* = 0.031). **B** Comparison of dust samples from flats (*n* = 16), houses (*n* = 6), a maternity hospital (NEO; *n* = 8), a pediatric hospital (ENT; *n* = 6), and a research center (RCX; *n* = 8) tested by Kruskal–Wallis ANOVA (*p* = 0.023) and by post hoc Dunn’s test (*p* > 0.05)

The analysis of environments revealed a higher alpha diversity of the dust bacteriome from workplaces compared to households (number of amplicon sequence variants (ASVs) and Chao1, both *p* ≤ 0.05); see Fig. 1A. In addition, dust from houses had higher bacterial alpha diversity than that from flats, although this relationship was of borderline significance (Dunn’s test, number of ASVs, *p* = 0.069; Kruskal–Wallis ANOVA, Shannon index, *p* = 0.072); dust from flats had the lowest bacterial diversity overall (Fig. 1B).

**Fig. 2:**
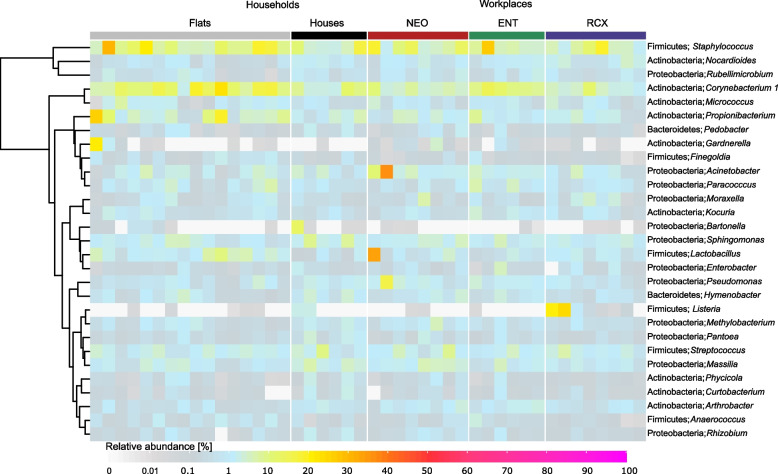
Bacterial composition at the genera level in individual indoor dust samples from households and workplaces. Each column represents one sample. Samples from households include flats (*n* = 16) and houses (*n* = 6); samples from workplaces include a maternity hospital (NEO; *n* = 8), a pediatric hospital (ENT; *n* = 6), and a research center (RCX; *n* = 8). All samples reached more than 20,000 reads per sample after 16S rRNA sequencing analysis. Only the 20 most abundant genera of bacteria from flats, houses, and workplaces separately were included in the heatmap, unassigned genera were excluded

Applying PERMANOVA to the bacterial composition at the genus level, a significant effect of the following variables on the dust bacteriome composition was observed: place (workplaces, households; q ≤ 0.05) and a combination of the type of households and different workplaces (Flats, Houses, NEO, ENT, RCX; q ≤ 0.05, Additional file 1). The bacterial composition of the 20 most abundant bacterial genera in dust samples from flats, houses, and workplaces (NEO, ENT, and RCX) is shown in Fig. 2. Despite the observed differences in alpha diversity, the number of unique genera in different places (flats, houses, NEO, ENT, RCX) was low (Additional file 4) and the comparison of relative abundances of individual genera between places revealed only a few taxa significantly differing in abundance between environments (Additional file 5): *Listeria*, *Corynebacterium 1*, *Prevotella*, *Anaerococcus*, *Peptoniphillus*, and *Lactobacillus* (q ≤ 0.05; Kruskal–Wallis ANOVA). The relative abundance of the *Listeria* genus was significantly higher in dust samples from the RCX workplace compared to other indoor environments (q = 0.039; Kruskal–Wallis ANOVA).

In flats, *Corynebacterium 1*, *Prevotella*, and *Lactobacillus* were more abundant than in houses. *Corynebacterium 1* and *Peptoniphilus* were found relatively more abundant in flats compared to the NEO workplace. In flats, *Lactobacillus* was more abundant compared to ENT; and *Corynebacterium 1*, *Prevotella*, *Anaerococcus*, and *Peptoniphilus* were more abundant compared to RCX. No differences in the 20 most abundant genera were observed in dust samples collected in NEO compared to samples from ENT (Additional file 5).

### Nasopharyngeal bacteriome

Overall, we found 803 genera representing 39 phyla in the nasopharyngeal microbiome, with the *Firmicutes* (median 36.5%) and *Actinobacteria* (median 20.0%) being the most abundant phyla, and *Staphylococcus* (median 17.1%) and *Corynebacterium 1* (median 9.2%) the most abundant genera.

**Fig. 3:**
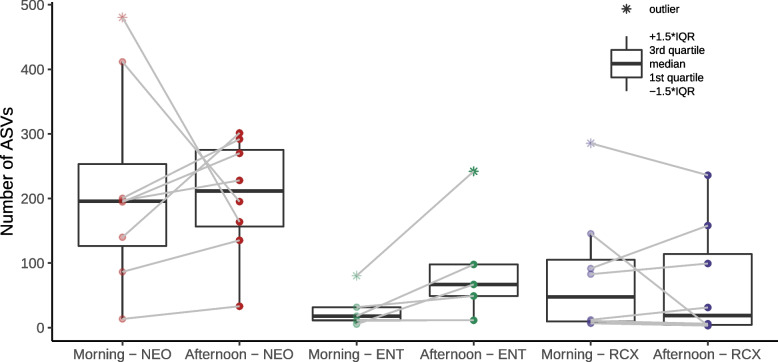
Comparison of alpha diversities (the number of ASVs) of nasopharyngeal swabs. Nasopharyngeal swabs collected in the morning and in the afternoon (after an 8 h exposure to the workplace environment) were grouped according to participants’ workplaces; maternity hospital (NEO; *n* = 8), pediatric hospital (ENT; *n* = 5), and a research center (RCX; *n* = 8). Morning and afternoon samples were compared using the Wilcoxon signed-rank test (*p* > 0.05). One nasopharyngeal sample (from a participant working at ENT) collected in the afternoon was excluded from the analysis due to the low DNA conent in the sample

The alpha diversities of nasopharyngeal bacteriome were similar between samples collected in the morning and in the afternoon from each participant (*p* > 0.05, Wilcoxon signed-rank test, see Fig. 3).

There was no association of the nasopharyngeal bacteriome with the participants’ ages and sexes (*p* > 0.05 for both, PERMANOVA). The composition of bacterial genera in both morning and afternoon nasopharyngeal samples was significantly associated with the workplace (NEO, ENT, and RCX, *p* ≤ 0.05 for both, PERMANOVA).

**Fig. 4:**
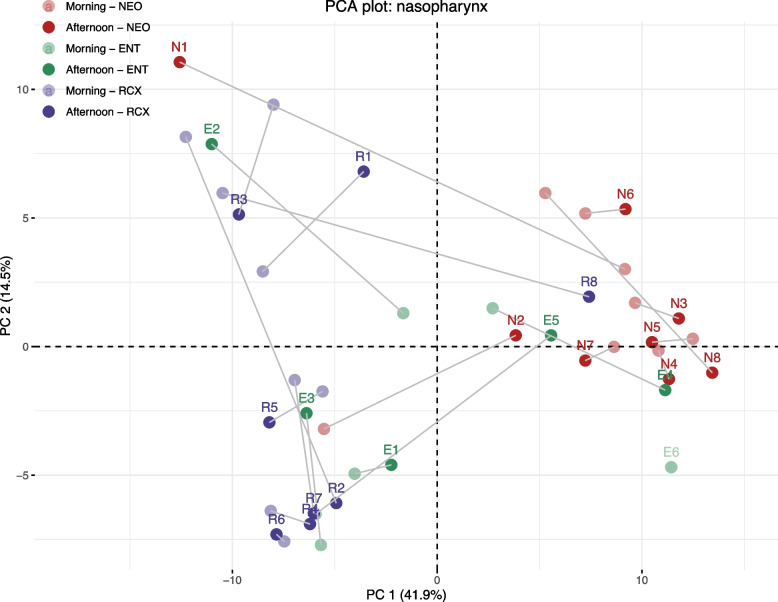
Principal component analysis of morning and afternoon nasopharyngeal swab samples based on their bacterial composition. Morning samples were collected in the households, afternoon samples were collected after an 8 h exposure to the workplace environment. The dots represent the samples color-coded according to the participants’ workplaces – maternity hospital (NEO/N; *n* = 8), pediatric hospital (ENT/E; *n* = 6), and the research center (RCX/R; *n* = 8). Paired samples from the same participant are connected with a grey line. One nasopharyngeal sample (E6) collected in the afternoon was excluded from the analysis due to the low DNA conent in the sample

**Fig. 5:**
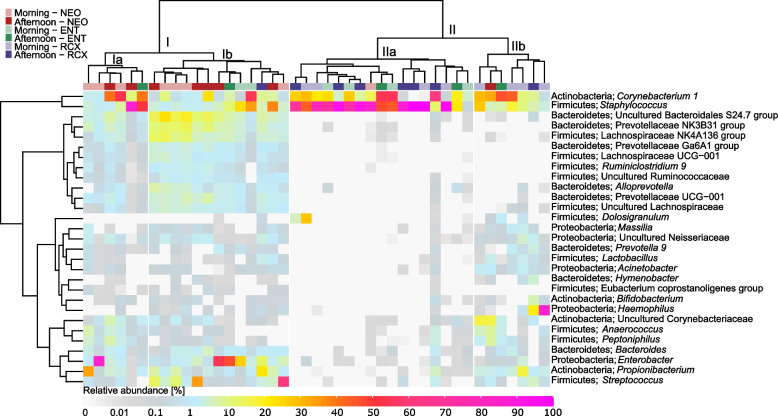
Bacterial composition at the level of genera in nasopharyngeal samples. Each column represents one sample. Two major clusters (I and II) were identified; the first cluster included particularly participants working at NEO, the other cluster consisted of participants working at RCX. Nasopharyngeal samples from ENT workers were distributed equally between both major clusters Samples were collected in the morning and in the afternoon (after an 8 h exposure to the workplace environment) from participants working at the maternity hospital (NEO; *n* = 8), pediatric hospital (ENT; *n* = 6), and a research center (RCX; *n* = 8). Only the 20 most abundant genera from each workplace were included in the heatmap. One nasopharyngeal sample collected in the afternoon was excluded from the analysis due to the low DNA conent in the sample

The composition of the morning and afternoon nasopharyngeal bacteriomes of the participants working at NEO, ENT, or RCX workplaces was visualized using the PCA plot, see Fig. 4. We observed that nasopharyngeal samples were distributed mostly according to the participant´s workplace – NEO or RCX. Similarly, unsupervised hierarchical clustering of the 20 most abundant genera found in nasopharyngeal samples of participants revealed two major clusters (Fig. 5). The first cluster consisted mainly of nasopharyngeal samples from participants working at NEO and the second cluster consisted mostly of participants working at RCX. Nasopharyngeal samples from ENT workers are distributed evenly between both major clusters.

**Fig. 6:**
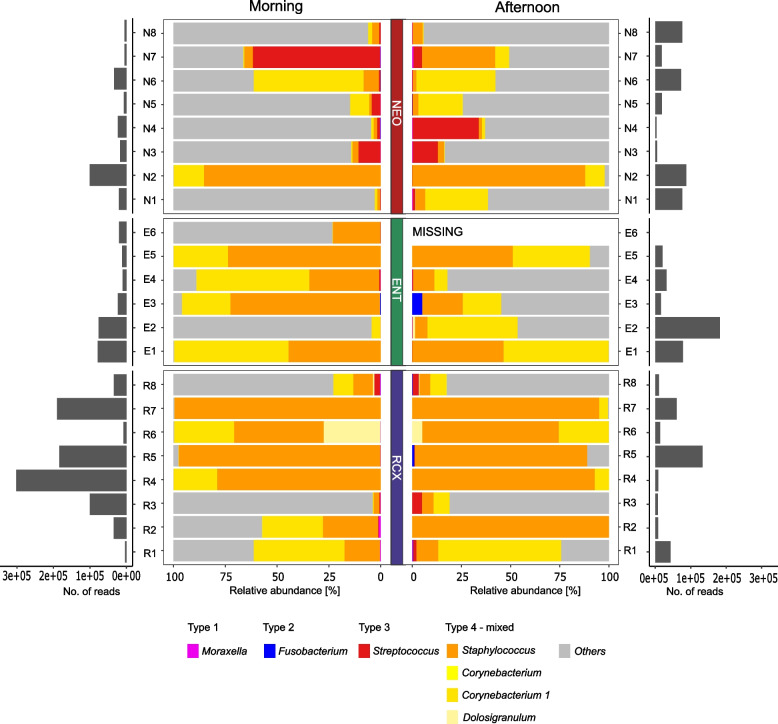
Relative abundances of bacterial genera defining four nasopharyngeal bacteriome types according to De Boeck et al. [60]. Samples were collected in the morning and in the afternoon (after an 8 h exposure to the workplace environment) and classified according to the participants’ workplaces – maternity hospital (NEO/N; *n* = 8), pediatric hospital (ENT/E; *n* = 6), and the research center (RCX/R; *n* = 8). One nasopharyngeal sample (E6) collected in the afternoon was excluded from the analysis due to the low DNA conent in the sample (MISSING). Left and right grey barplots show the numbers of reads in the respective samples

Some nasopharyngeal samples had a low number of reads (< 10,000 reads per sample); some were dominated by one or two bacteria (*Staphylococcus* and/or *Corynebacterium 1*), see Fig. 6. The classification of nasopharyngeal bacteriome types according to De Boeck et al. [60] showed that only type 4 (mixed *Corynebacterium*, *Staphylococcus*, and *Dolosigranulum* type) and type 3 (*Streptococcus*) were identified in our study. The proportion of genera defining nasopharyngeal types was similar in participants working in ENT and RCX, while that of NEO workers was more diverse and differed from the other workplaces, see Fig. 6.

The comparison of individual bacterial genera in the afternoon nasopharyngeal samples among participants from different workplaces (NEO, ENT, RCX) showed significant differences for unadjusted p-values only (q > 0.05, *p* ≤ 0.05). A trend was observed for the increased/decreased abundances of *Staphylococcus* and *Corynebacterium 1* in nasopharyngeal samples from the RCX workplace compared to the other workplaces but was borderline insiginificant (*p* = 0.057 and *p* = 0.062, respectively, Kruskal–Wallis ANOVA) and higher abundance of *Streptococcus* in the nasopharyngeal bacteriome of NEO workers compared to participants from other workplaces (*p* = 0.302, Kruskal–Wallis ANOVA, Additional file 5).

### Comparison between indoor dust and nasopharyngeal bacteriome

**Fig. 7:**
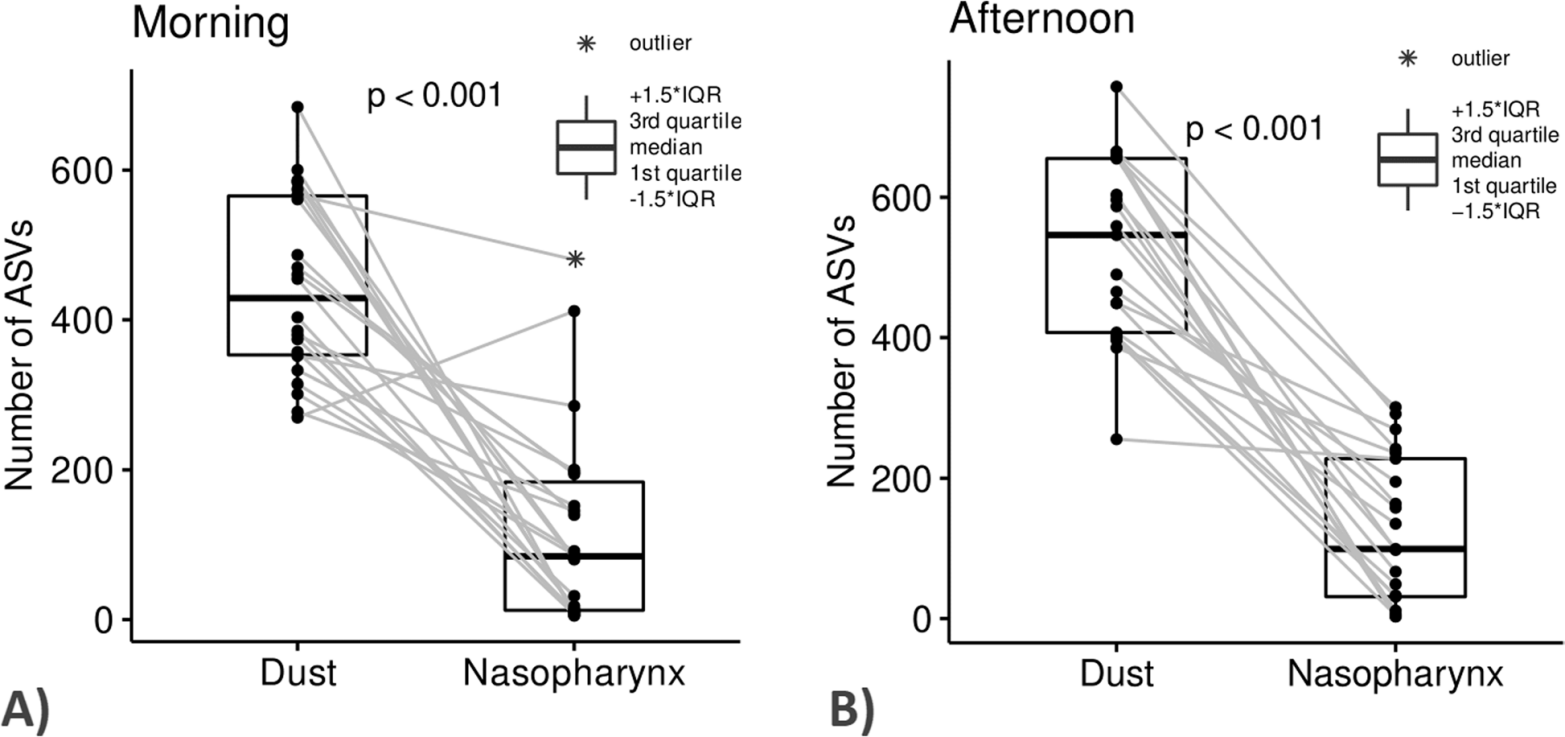
Comparison of alpha diversities (the number of ASVs) in dust and nasopharyngeal samples. The paired boxplots show (**A**) 22 indoor dust samples from households and the paired nasopharyngeal swabs collected in the morning and (**B**) 21 indoor dust samples from workplaces and paired nasopharyngeal swabs collected in the afternoon (i.e., after an 8 h exposure to workplace environment). Differences between matrices were tested using the Wilcoxon signed-rank test (*p* < 0.001). One nasopharyngeal sample collected in the afternoon was excluded from the analysis

The alpha diversity of the dust bacteriome was significantly higher than that of nasopharyngeal bacteriome in all tested alpha diversity metrics (all *p* < 0.001, Wilcoxon signed-rank test), see Fig. 7.

In contrast to nasopharyngeal samples, *Cyanobacteria* were highly abundant in dust samples (medians of 0.2%, 20.2%, respectively). The phylum *Firmicutes* was detected in all samples, regardless of the sample type. While only 1 phylum (*Firmicutes*) was present in all nasopharyngeal swabs, 13 phyla were found in all dust samples. The chloroplast *16S rDNA* sequence was the most abundant in the dust samples (median 18.9%; Additional file 3), while its relative abundance in the nasopharyngeal swabs was low (median 0.08%).

Two bacterial genera, *Staphylococcus* and *Corynebacterium 1,* were the most abundant in both matrices. The median relative abundance of *Propionibacterium*, *Acinetobacter*, *Streptococcus*, *Sphingomonas*, *Massilia*, and *Lactobacillus* exceeded 1% in the dust samples.

Representations of two bacterial genera (*Sphingomonas* and *Porphyromonas*) positively correlated between the morning nasopharyngeal samples and dust samples from households (*r* = 0.59 and *r* = 0.53, respectively, *p* ≤ 0.05, q ≥ 0.05, in both). A positive correlation between the representation of bacteria in the afternoon nasopharyngeal swabs and dust samples from workplaces was observed for eight bacterial genera (see Table 1).

**Table 1:** Correlations of the representation of bacterial genera between indoor dust samples and nasopharyngeal swabs

Sampling	Type of samples	Genus	r_S_	*p*	q
Morning	Np vs. DH	*Sphingomonas*	0.588	0.005	0.284
		*Porphyromonas*	0.532	0.012	0.353
Afternoon	Np vs. DW	*Bacillus*	0.578	0.007	0.357
		*Eremococcus*	0.519	0.017	0.357
		*Subdoligranulum*	0.512	0.019	0.357
		*Clostridium *sensu stricto* 1*	0.506	0.02	0.357
		*Granulicatella*	0.474	0.031	0.423
		*Dolosigranulum*	0.444	0.045	0.471
		*Enterobacter/Klebsiella*	0.535	0.014	0.357
		*Neisseriaceae* uncultured	0.471	0.032	0.423

Samples were collected from 21 participants in the morning at their households and in the afternoon at their workplaces (after an 8 h exposure to workplace environment; only statistically significant bacterial genera are listed)

*Np* nasopharyngeal swab, *DH* dust samples collected at households, *DW* dust samples collected at workplaces. *Klebsiella* and *Enterobacter* cannot be distinguished based on bioinformatics processing due to their similar genetic sequences

**Fig. 8:**
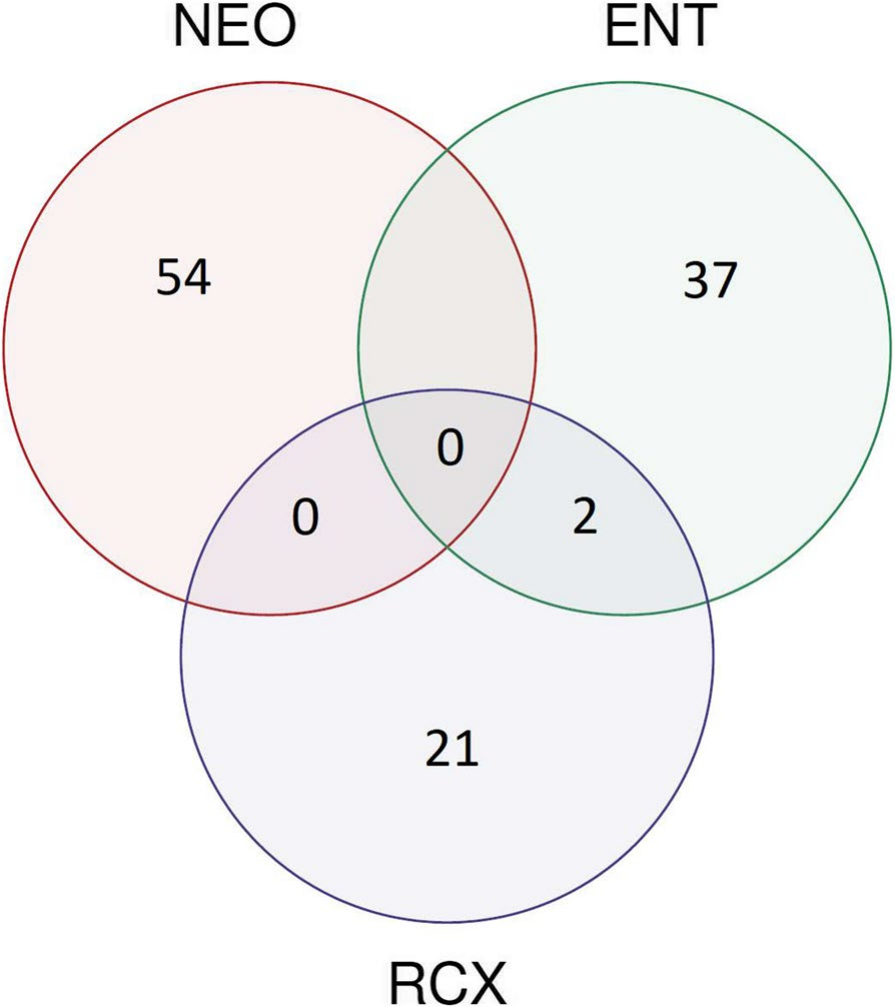
Venn graph indicating enrichment of the nasopharyngeal bacteriomes from indoor dust bacterial genera. The enrichement was evaluated in 21 participants after the 8 h exposure to the workplace environment and overlaps among workplaces – maternity hospital (NEO; *n* = 8), pediatric hospital (ENT; *n* = 6), and research center (RCX; *n* = 8)

To determine whether the workplace dust bacteriome influences the composition of the nasopharyngeal bacteriome of individuals, we looked for bacteria that were not present in the morning nasopharyngeal samples but were found in both workplace dust and afternoon nasopharyngeal samples. After the 8 h spent at the workplace, 76% (*n* = 16) of nasopharyngeal samples were enriched with at least one new bacterial genus from the workplace dust (100% in NEO; 80% in ENT, and 50% in RCX group). There was an overlap in the “enriching” bacterial genera between hospital workplaces (NEO and ENT). The overlap between hospital and research center was minimal (only two genera overlapped between ENT and RCX, no overlap with NEO was observed), see Fig. 8. Genera that enriched the nasopharyngeal bacteriome in at least two participants from each workplace are shown in Table 2.

**Table 2:** Bacterial genera from the workplace indoor dust enriching the nasopharyngeal bacteriome

Workplace	Genus
NEO	*Alistipes*
	*Pseudoclavibacter*
	*Cloacibacterium*
	*Ruminococcus 2*
	*Planctomyces*
	*Cellvibrio*
ENT	*Eubacterium coprostanoligenes*
RCX	*Burkholderia*

Samples from 21 participants were evaluated after an 8 h exposure to the workplace environment. Only genera observed in at least 2 participants from each workplace (ENT, NEO, RCX) are shown in the table

## Discussion

To date, research on the indoor bacteriome has focused mostly on specific environments such as hospitals [27–30] or households of families suffering from asthma and other chronic diseases [17, 18]. However, there is a lack of information about the impact of dust bacteriomes from households and workplaces, including hospitals and office buildings, on human bacteriomes.

Our pilot study is the first to analyze indoor dust bacteriomes from both households and workplaces simultaneously with the nasopharyngeal bacteriomes of individuals living or working in the evaluated buildings. This pilot study was intended to provide primary data for the Central European Longitudinal Study of Parenthood and Childhood: The Next Generation (CELSPAC: TNG) cohort study, which is designed as a new prospective birth cohort that will follow up 7,000 children from their prenatal period to adolescence with the aim of assessing environmental factors potentially affecting children's health. The CELSPAC: TNG study is based on previous research at Masaryk University, especially on the Czech part of the WHO-initiated European Longitudinal Study on Pregnancy and Childhood (ELSPAC) [61].

The maternity ward is usually the first environment that newborns encounter after their birth and that can influence their health and microbiome. The next environment they are exposed to are their homes. Children often play on the floor, which makes dust from the household floors another possible source of microorganisms. The children's hospital, specifically the ENT department, is another high-risk place where children can be exposed to microorganisms that can affect their health. The RECETOX workplace was selected as a representative of an “office” type working environment.

We are aware that the design of the pilot study does not allow a precise evaluation of some additional data (humidity, sampling date, room temperature, chronic airway diseases, allergies, BMI, etc.) as the groups were not randomized and have not a balanced number of samples; however, we believe that they can still point to variables that should be taken into account in larger studies.

### Indoor dust bacteriome

In our pilot study, a higher bacterial diversity was observed in the dust samples from workplaces than from households. The examined workplaces were commercially used buildings characterized by higher levels of human occupancy and usage of HVAC (heating, ventilation, air conditioning) systems, both contributing to the higher abundance of microorganisms and their spread in the building [7–9, 16]. If investigating other types of workplaces with different indoor conditions, the results would likely be different. The same is true for households, where there may be large differences even between individual houses, where (for example) the pet microbiomes may be a major factor in addition to human occupancy [16, 62, 63].

Previous studies have observed the possible effects of the outdoor environment on the indoor bacteriome [5, 6, 64]. Our data showed significantly higher bacterial diversities in the dust samples from houses compared to flats. Flats are often located on higher floors of buildings, where lower bacterial diversity was observed in another study compared to lower floors [6], which are typical of family houses. Furthermore, family houses are usually located on the outskirts of the city, in greater contact with plant and soil bacteria from the surrounding nature (gardens, forests), which has been reported to be associated with higher bacterial diversity [38]. Additionally, we observed effects of sampling date, humidity, and room temperature on the dust bacteriome, as was described before [8, 17].

In our study, *Staphylococcus* and *Corynebacterium* were the most abundant genera in the indoor dust samples, which is consistent with the results reported by Hanson et al. [12]. According to other studies, these Gram-positive bacteria predominate in the indoor dust; in contrast, Gram-negative bacteria are more often detected in dust from the outdoor environment [7, 12].

### Nasopharyngeal bacteriome

To the best of our knowledge, this is the first study evaluating the nasopharyngeal bacteriome of people before they went to work and after 8 h of exposure to the workplace environment. No significant change in the bacterial composition of nasopharyngeal samples taken before and after 8 h exposure to the workplace environment was observed. The nasopharyngeal bacterial composition seemed to be quite stable during the day.

The structure of the nasopharyngeal bacteriome in workers at the maternity hospital (NEO) was more diverse with high abundances of bacteria related to the gut bacteriome (*Bacteroidales, Prevotellaceae, Lachnospiraceae, and Ruminococcaceae*) than in participants from the pediatric hospital (ENT) and the research center (RCX). Although these differences in study groups were observed, the nature of our study did not allow us investigate if the workplace did in fact shape the nasopharyngeal bacteriome richness (over longer periods of time) and caused these differences between individuals from different workplaces.

In some samples, a very high number of reads dominated by only one or two bacteria (*Corynebacterium 1* and or *Staphylococcus*) was detected. Such a low bacterial diversity in human samples, which are usually highly abundant for bacterial DNA, is generally considered pathological. A decrease in microbial diversity, richness and evenness is a frequent feature in chronic inflammatory diseases such as in patients with chronic rhinosinusitis [65]. On the other hand, the number of reads in some samples was very low, which could be a consequence of contamination with chemicals, such as disinfectants used in hospitals, etc.

In line with our findings, *Staphylococcus* and *Corynebacterium* genera were previously described as highly abundant in the nasopharynx [41, 60, 66]. De Boeck et al. [60] suggested that the nasopharyngeal bacteriome of healthy adults can be classified into 4 profiles according to the most dominant bacterial genera*:* (1) *Moraxella* type, (2) *Fusobacterium* type, (3) *Streptococcus* type, (4) Mixed *Corynebacterium*, *Staphylococcus*, and *Dolosigranulum* type. In our study, only the Mixed type and *Streptococcus* type were observed. While the Mixed *Corynebacterium, Staphylococcus,* and *Dolosigranulum* type was present mainly in the workers from ENT and RCX, NEO participants had a different structure of the nasopharyngeal bacteriome with a higher abundance of the genus *Streptococcus* and other genera compared to other participants. Previously, representatives of the genus *Streptococcus* have been reported to be associated with a higher risk of respiratory tract infections [67, 68].

Contrary to a study by Whelan et al. [51], we were not able to see any difference in age groups, probably due to the small number of participants. For the same reason, we could not evaluate the effects of other variables, such as chronic airway diseases, allergies, and BMI.

### Comparison of the dust bacteriome and nasopharyngeal bacteriome

We revealed significantly higher alpha diversity of the dust bacteriome (median 351) compared to the nasopharyngeal bacteriome (median 57). This is consistent with previous studies reporting the number of bacterial ASVs in indoor dust samples to be as high as 48,500 [69] compared to < 100 in nasopharyngeal mucosa [70, 71].

Positive correlations between the abundances in the dust and nasopharyngeal samples were observed for several bacterial genera both in the morning (with household dust) and afternoon (workplace dust). This suggests a possible relationship between the dust and the nasopharyngeal bacteriome. Bacteria from the dust are reaching the nasopharynx and, therefore, meet the first condition or being able to colonize the nasopharyngeal mucosa; the possible success of such colonization was, nevertheless, not investigated in our study. Although some anaerobic bacteria were detected in the nasopharynx, it does not mean that these bacteria survive and colonize the upper respiratory tract in the long term. Similar to dust particles and other pollutants in the air, various microorganisms, living or dead, can enter the respiratory tract.

Moreover, we evaluated the enrichment of the nasopharynx with new bacterial genera that were not present in the morning swabs but appeared in the afternoon nasopharyngeal swabs after spending 8 h at the workplace. This enrichment of the nasopharyngeal bacteriome was observed in 76% of the study participants. Interestingly, all workers from the maternity hospital NEO had their nasopharyngeal bacteriome enriched with at least one bacterial genus from the workplace dust in the afternoon compared with their morning samples.

Our study has some limitations. The number of participants from 3 different workplaces is low; however, this was a pilot study, and the number was sufficient to obtain pilot data. In our study, negative controls were not sequenced, which could be problematic, especially in the interpretation of the findings in human samples; however, our aim was not to describe the bacteriome of the nasopharynx but to perform a paired comparison of bacteriomes from the nasopharynx and to investigate changes in nasopharyngeal samples after exposure to the indoor environment.

Also the nasopharyngeal resistome as well as virome has received a lot of attention in recent years, especially after the COVID-19 pandemic. However, the environmental virome and its potential relationship to the human respiratory virome is still not fully understood, which could provide valuable information on the spread of viruses in the external environment.

## Conclusions

In this pilot study, we investigated the dust bacteriome from households and workplaces and its impact on the human nasopharyngeal bacteriome. The analysis of the dust bacteriome revealed that the bacterial diversity was significantly higher in workplaces than in households. The most diverse nasopharyngeal bacteriome was observed in participants from the maternity hospital in comparison to those from the pediatric hospital and the research center. Interestingly, the nasopharyngeal bacteriomes in most study participants were enriched with new bacterial genera after the 8 h exposure to the workplace environment; however, the overall composition of the nasopharyngeal bacteriome remained relatively stable during the day.

Our results show the influence of the indoor environment where people live or work and its impact on the human nasopharyngeal bacteriome; however, other environmental factors can be involved in the formation of the human respiratory microbiome and further research evaluating this issue is needed.

## Materials and methods

### Study design

This pilot study was designed as a cross-sectional study observing a defined population and intended to provide primary data for the CELSPAC: TNG cohort study, which is studying children’s health from the birth onwards and the factors that affect it throughout their life course. Exposure to the indoor environment was evaluated together with the outcome of this exposure, i.e., the changes in the nasopharyngeal bacteriome. Samples from the nasopharynx of a specific population and the indoor dust were collected, and their bacteriomes were analyzed. The study was conducted in 2018 (May to August) prior to the COVID-19 pandemic and none of the participants wore a mask. Ethics approval for this study was granted by the (C)ELSPAC Ethics Committee of Masaryk University. Written informed consent was obtained from all study participants in accordance with the Declaration of Helsinki.

Inclusion criteria for this study were: age ≥ 18 years, willingness to participate in the study, living in Brno, Czech Republic (within a radius of 25 km), working at the i) maternity hospital – Department of of Gynaecology and Obstetrics (Neonatology, NEO), University Hospital Brno, ii) pediatric hospital – Department of Pediatric Otorhinolaryngology (Ear, Nose, and Throat, ENT), University Hospital Brno, or iii) the research center – RECETOX (RCX) at Masaryk University, Brno. Volunteers living > 25 km from their workplace and those who had a concomitant acute illness of the respiratory tract were excluded from this study.

### Sample and data collection

The indoor dust samples from participants’ households and workplaces were collected at the same time points (i.e., household dust in the morning and workplace dust in the afternoon, after the participant’s 8 h exposure to the workplace environment) as nasopharyngeal samples. Dust samples from households and workplaces were collected using a conventional vacuum cleaner with a sampling head specifically designed for our purposes [72] (Additional file 6). The dust was captured on QM-A Quartz Microfiber filters (Whatman, UK; diameter 101.6 mm, pore size 2.2 μm) placed inside the sampling head. Before each sampling, the sampling head was washed with 98% ethanol and the filters were autoclaved in BKM-Z24B (Biobase, CHN; temperature 134°C, time 360 s, additional drying 600 s, 3 vacuum pulses). The dust was collected from 50% of the floor in the participant’s bedroom and from 50% of the office floor at the workplace where the participant spent most of his/her working hours. The size of the vacuumed area, the room temperature, and the humidity of the environment were measured. Dust samples were immediately inserted into a temperature-controlled portable freezer tempered at -20 °C (CoolFreeze CF-11, Dometic Waeco International GmbH, Emsdetten, Germany), transported to the laboratory, and stored at -20°C. The temperature during transport was monitored using a temperature logger TESTO 175 T1 (Testo, Germany).

Nasopharyngeal swabs from each participant were collected in the morning before the participant set off for work and in the afternoon at the end of their working hours (i.e., after an 8 h exposure to the workplace environment). Samples were taken by trained doctors from the Department of Pediatric Otorhinolaryngology, University Hospital Brno. Nasopharyngeal swabs were collected using nylon FLOQSwabs 553C (COPAN, CA, USA) by rotating them ten times against the nasopharyngeal wall. Similarly to the dust samples, each swab sample was immediately transferred into a temperature-controlled portable freezer tempered at -20 °C (CoolFreeze CF-11, Dometic Waeco International GmbH, Emsdetten, Germany), transported to the laboratory, and stored at -20°C. The morning nasopharyngeal sample from each participant represented the baseline, which was compared to the afternoon nasopharyngeal sample. All study participants were transported to their work/home by our technician or used their car to eliminate the effects of public transport. In all cases, the journey to and from the workplace took less than 30 min.

Information about each participant’s health status and description of the household environment (type of household, information about residents, etc.) was collected via questionnaire (Additional file 7).

### DNA extraction

The filter with captured dust was homogenized using Mixer Mill MM 301 (Retsch GmbH, GER; frequency 25 s^−1^, time 30 s). DNA was extracted from 200 mg of the homogenate utilizing the DNeasy PowerLyzer PowerSoil Kit (QIAGEN, GER). The dust was weighed into a Bead Tube (part of the extraction kit) and 750 μl of the Bead Solution and 60 μl of prewarmed (64 °C) C1 solution from the kit were added. The mixture was homogenized using BeadBlaster 24 (Benchmark Scientific, USA; 6,5 M/s, 45 s). Samples were centrifuged in Sigma 1–14 (Sigma, GER; 1 min, 10 000 RCF), the supernatant was transferred into Sample Tube RB (accessories for the QIAcube device, QIAGEN, GER), and 1 μl of RNase (New England BioLabs, USA; 25 μg/ml) was added to the supernatant. Subsequent steps were performed using the robotic workstation QIAcube (QIAGEN, GER) according to the manufacturer’s instructions for DNeasy PowerLyzer PowerSoil program; the elution volume was 50 μl.

DNA from nasopharyngeal swabs was extracted manually with the QIAamp DNA Mini Kit (QIAGEN, GER) according to the manufacturer’s instructions, the elution volume was 30 μl.

The purity and concentration of extracted DNA from dust and nasopharyngeal samples were determined using a spectrophotometer NanoDropND-1000 (Thermo Fisher Scientific, USA), and the quality of DNA was assessed using gel electrophoresis. Extracted DNA was stored at -20°C.

### *16S rDNA* gene analysis

Extracted DNA was used as a template in amplicon PCR to target the V4 hypervariable region of the bacterial *16S rDNA* gene. PCR amplification was performed using the primer pair consisting of Illumina overhang nucleotide sequences, an inner tag, and gene-specific sequences. The Illumina overhang served to ligate the Illumina index and adapter. Each inner tag, i.e., a unique sequence of 12 bp, was designed to differentiate samples into groups. DNA was amplified utilizing the polymerase Q5 HighFidelity 2 × MM (New England BioLabs, USA). PCR reactions were carried out in a total volume of 30 μl with the following conditions: initial denaturation 98 °C/5 min, 30 cycles of 98 °C/10 s, 55 °C/15 s, 72 °C/25 s, and final extension 72 °C/2 min. Negative and positive controls were included for PCR amplification. The amplified PCR products were determined by gel electrophoresis and subsequently purified using Agencourt AMPure XP Beads (Beckman Coulter, USA).

Samples with different inner tags were equimolarly pooled based on fluorometrically measured concentrations using a fluorometer Synergy HTX (BioTek, USA) and a Quant-iT™ dsDNA Assay Kit high sensitivity (Thermo Fisher Scientific, USA). Pools were used as templates for a second PCR with Nextera XT indexes (Illumina, USA). Differently indexed samples were equimolarly pooled based on fluorometrically determined concentrations as before. The prepared library was checked on a 2200 TapeStation Instrument (Agilent Technologies, USA) using Agilent D1000 ScreenTape System Kit (Agilent Technologies, USA). The final library was diluted to a concentration of 8 pM and 20% of PhiX DNA (Illumina, USA) was added. Sequencing was performed using the Miseq reagent kit V2 (500 cycles; 2 × 250 bp) on a MiSeq instrument according to the manufacturer’s instructions (Illumina, USA).

### DNA sequence analysis

Paired-end reads from 16S rRNA sequencing were processed as follows. Preprocessing steps included the trimming of low-quality 3’ ends of reads, removal of pairs of reads containing N base, and removal of pairs containing very short reads. In order to minimize sequencing and PCR-derived errors, forward and reverse reads were denoised using the DADA2 amplicon denoising R package [73]. Following denoising, the forward and reverse reads were joined into a single longer read using the fastq-join read joining utility [74]. To be joined, reads in pairs had to overlap in at least 20 base pairs with no mismatches allowed. Pairs in which this was not the case were discarded. As the final step, chimeric sequences were removed from the joined reads using the remove Bimera function of the DADA2 R package. Subsequent taxonomic assignment was conducted by the uclust-consensus method from the QIIME [75] microbial analysis framework using the Silva v. 123 reference database [76]. The sequencing data was uploaded into the Sequence Read Archive (NCBI) under the accession number PRJNA788869; the ASV table is in the supplementary file (Additional file 8).

### Statistical analysis

Differences in alpha diversity (estimated by the Shannon, Simpson, and Chao1 diversity indexes, and the number of ASVs) between various groups were statistically evaluated using the Mann–Whitney *U* test or Kruskal–Wallis ANOVA, followed by a post hoc Dunn’s test.

Statistical analysis of the bacterial composition was performed at the genus level. The Chloroplast reads and unassigned reads were excluded from further analyses. Only taxa with an abundance of ≥ 0.5% of minimal sequencing depth (20,119 reads for dust and 3537 reads for nasopharyngeal samples) in at least three samples were included in the subsequent analysis to avoid high sparsity in data. This resulted in 161 genera for dust samples and 115 genera for nasopharyngeal samples. Sample rarefaction curves were plotted on both unfiltered and filtered data using rarecurve function of R package vegan v. 2.6.4. and can be found in Additional file 9. Prior to statistical analysis, data were treated as compositional and transformed using the centered log-ratio (CLR) transformation [77]. All zeroes in the original data were replaced using the count zero multiplicative method [78]. Depending on the type of analysis, taxa filtering was performed (i) separately for dust and nasopharyngeal samples and (ii) for all samples combined.

Differences and variability in the bacterial composition of the top 20 genera in individual matrices (dust, nasopharynx) were first visualized using multivariate approaches, namely the Principal Component Analysis (PCA) and heatmaps based on hierarchical clustering (Euclidean distance, Ward’s linkage method). The permutational multivariate analysis of variance (PERMANOVA) was used to test differences in the dispersion and centroids of the groups of bacterial communities, based on a Euclidean distance. *P*-values were calculated as the mean values of 1,000 repetitions (with 999 permutations, see Additional file 1). Kruskal–Wallis ANOVA with Dunn’s post-hoc test or Mann–Whitney *U* test were used to compare the differences in abundances of taxa between the groups. Wilcoxon signed-rank test was used to compare paired (nasopharyngeal morning and afternoon) samples. Spearman’s rank correlations were calculated to determine the relationships between dust and nasopharyngeal samples. The resulting p-values were adjusted for multiple hypotheses testing using the Benjamini–Hochberg procedure (BH). Results were considered significant at FDR ≤ 0.05. All statistical analyses were performed in R (v. 4.1.1) [79] using additional R packages: genefilter (v. 1.72.1) for taxa filtering [80]; PERFect (v. 1.4.0) for permutation filtering [81]; zCompositions (v. 1.3.4) for zero replacement [82]; compositions (v. 2.0–2) for CLR transformation [83]; vegan (v. 2.5–7) for PERMANOVA [84]; gplots (v. 3.1.1) [85] and UpSetR (v. 1.4.0) [86] for visualization of bacteria intersections in different sample matrices; FDRestimation (v. 1.0.0) [87] for Benjamini–Hochberg correction; ggpubr (v. 0.4.0) for box and whiskers plots, barplots [88], ComplexHeatmap (v. 2.7.11.) for heatmaps [89] and limma (v. 3.46.0) [90] for Venn diagram.

## Supplementary Information

**Additional file 1. **Additional results.

**Additional file** **2.** Bacterial phyla in the dust and nasopharyngeal samples. * Number of occurrences of the specific phylum in the dust (n = 44)/nasopharyngeal samples (*n* = 44). One nasopharyngeal sample collected in the afternoon was excluded from the analysis. ** Values are based on the relative abundances of bacterial phyla in the dust/nasopharyngeal samples. One nasopharyngeal sample collected in the afternoon was excluded from the analysis.

**Additional file 3.** Bacterial genera in the dust and nasopharyngeal samples. * Number of occurrences of the specific genus in the dust (*n* = 44)/nasopharyngeal samples (*n* = 44). One nasopharyngeal sample collected in the afternoon was excluded from the analysis. ** Values are based on the relative abundances of bacterial genera in the dust/nasopharyngeal samples.

**Additional file 4. **UpSetR plot. Visualization of bacterial genera intersections in dust and nasopharyngeal samples. UpSetR plot shows the presence of specific bacterial genera across tested groups in nasopharyngeal and dust samples. The UpSetR plot distinguishes unique and shared taxa. Genera with a group median value of at least three reads were defined as present in the group. Unassigned taxa were not involved.

**Additional file 5.** Results of differential abundance testing between groups. Only genera with at least unadjusted *p*-value ≤ 0.05 are displayed. Specific groups and statistical tests used are summarized on the Overview sheet.

**Additional file 6.** Dust sampling head.

**Additional file 7.** The questionnaire for study participants.

**Additional file 8.** ASV table nonchimeric. Np, nasopharyngeal sample; D, dust sample; Mor, morning samples; Aft, afternoon samples; NEO, maternity hospital (*n* = 8); ENT, pediatric hospital (*n* = 6); RCX, research center (*n* = 8).

**Additional file 9.** Sample rarefaction curves plotted on both unfiltered and filtered data using rarecurve function of R package vegan v. 2.6.4.

## Data Availability

Sequencing data are available at the Sequence Read Archive (NCBI) under the accession number PRJNA788869.

## Abbreviations

ENT: Pediatric hospital
NEO: Maternity hospital
RCX: Research center
DADA2: Divisive amplicon denoising algorithm 2
QIIME: Quantitative insights into microbial ecology
ASV: Amplicon sequence variant
ANOVA: Analysis of variance
CLR: Centered log-ratio
PCA: Principal component analysis
PERMANOVA: Permutational multivariate analysis of variance
BH: Benjamini-hochberg procedure
q: Adjusted value
p: Unadjusted value
IQR: Interquartile range
