# Supplementary material for: Enrichment of human nasopharyngeal bacteriome with bacteria from dust after short-term exposure to indoor environment: a pilot study
Source: BMC Microbiol. 2023 Jul 31;23:202. doi: 10.1186/s12866-023-02951-5 (PMC10391871; doi:10.1186/s12866-023-02951-5)
Supplement: Supplementary file 1 — Additional file 1. Additional results. [file 12866_2023_2951_MOESM1_ESM.pdf]

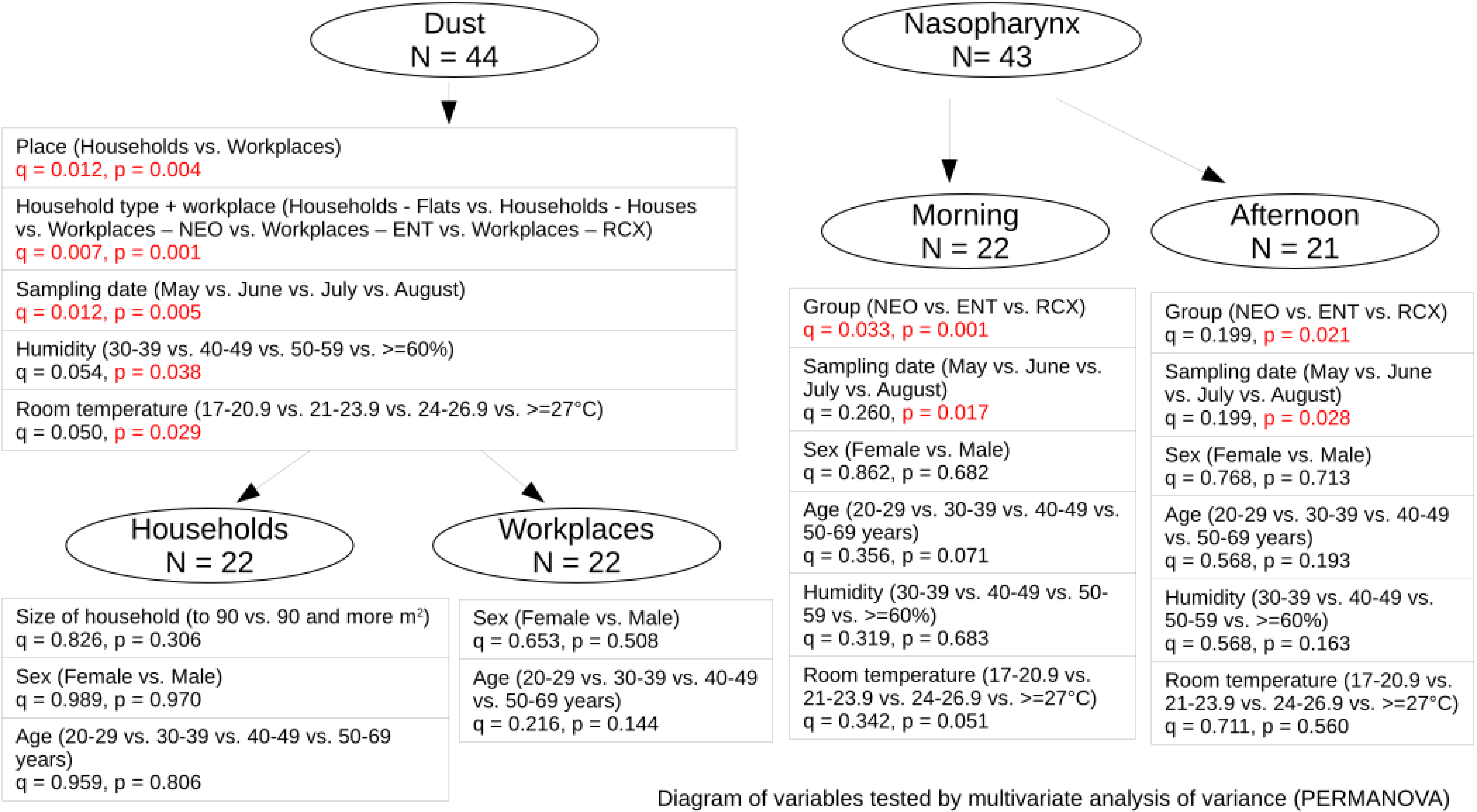

Diagram of variables tested by multivariate analysis of variance (PERMANOVA)
