# Supplementary material for: Enrichment of human nasopharyngeal bacteriome with bacteria from dust after short-term exposure to indoor environment: a pilot study
Source: BMC Microbiol. 2023 Jul 31;23:202. doi: 10.1186/s12866-023-02951-5 (PMC10391871; doi:10.1186/s12866-023-02951-5)
Supplement: Supplementary file 4 — Additional file 4. UpSetR plot. Visualization of bacterial genera intersections in dust and nasopharyngeal samples. UpSetR plot shows the presence of specific bacterial genera across tested groups in nasopharyngeal and dust samples. The UpSetR plot distinguishes unique and shared taxa. Genera with a group median value of at least three reads were defined as present in the group. Unassigned taxa were not involved. [file 12866_2023_2951_MOESM4_ESM.pdf]

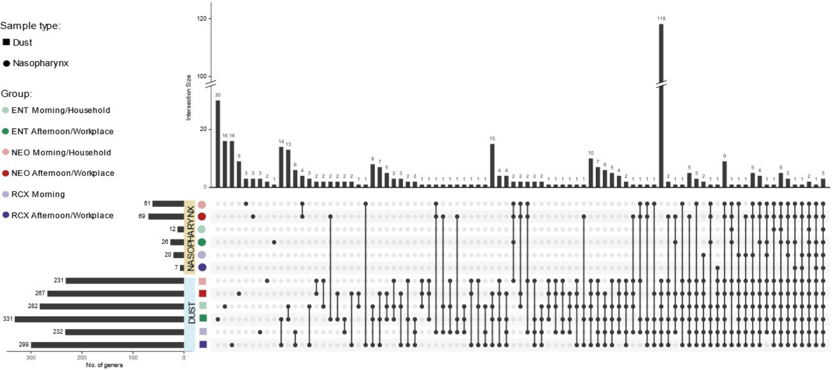

Sample type:
Dust
Nasopharynx
Group:
ENT Morning/Household
ENT Afternoon/Workplace
NEO Morning/Household
NEO Afternoon/Workplace
RCX Morning
RCX Afternoon/Workplace
Intersection Size
120
100
20
0
NASOPHARYNX
DUST
61
69
12
26
20
7
231
267
282
331
232
299
300
200
100
0
No. of genera
