# Supplementary material for: Enrichment of human nasopharyngeal bacteriome with bacteria from dust after short-term exposure to indoor environment: a pilot study
Source: BMC Microbiol. 2023 Jul 31;23:202. doi: 10.1186/s12866-023-02951-5 (PMC10391871; doi:10.1186/s12866-023-02951-5)
Supplement: Supplementary file 6 — Additional file 6. Dust sampling head. [file 12866_2023_2951_MOESM6_ESM.docx]

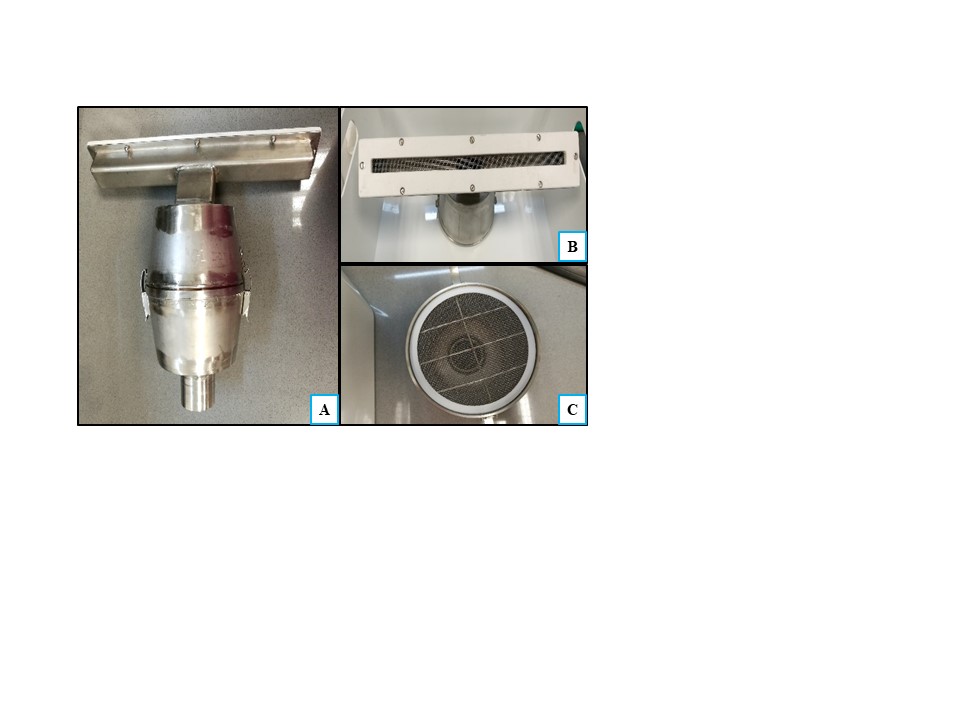


**Additional file 6.** Dust sampling head. A) General view of the sampling head, the head is made of stainless steel and PTFE and consists of several parts. B) The front of the head is in contact with the surface to be vacuumed and contains a 1 mm grid to prevent larger objects from being vacuumed. C) In the conical part of the head there is a circular grid on which a filter for dust collection is placed. This part of the head can be opened for easier handling of the filter and sterilization of the head.
