# Supplementary material for: Enrichment of human nasopharyngeal bacteriome with bacteria from dust after short-term exposure to indoor environment: a pilot study
Source: BMC Microbiol. 2023 Jul 31;23:202. doi: 10.1186/s12866-023-02951-5 (PMC10391871; doi:10.1186/s12866-023-02951-5)
Supplement: Supplementary file 7 — Additional file 7. The questionnaire for study participants. [file 12866_2023_2951_MOESM7_ESM.docx]

**DUST project questionnaire**

Your ID code:

**Please complete the following information**

Date of sample collection:

1. Gender: 🞎 Female 🞎 Male
2. Age:
3. Weight:
4. Height:
5. Your highest completed education:

🞎 Primary

🞎 Secondary education (including apprenticeship and university)

🞎 University degree

1. What district of Brno do you live in? ………………………………………..
2. What is the type of your household?

🞎 Flat

🞎 Family house

1. How many people live in your household? ………
2. What is the approximate age of the apartment/house you now live in?

🞎 Within 5 years

🞎 5-10 years

🞎 Older than 10 years

1. How many years have you lived here?

🞎 Less than a year

🞎 2 years

🞎 3 years

🞎 More than 3 years

1. What is the living area of the apartment you live in?

🞎 Less than 50 m2

🞎 50 - 70 m^2^

🞎 70 – 90 m^2^

🞎 More than 90 m^2^

1. Is there a garage in the house you live in?

🞎 Yes

🞎 No

1. Is the house insulated?

🞎 Yes → If yes:

What type of insulation does your house have (external walls)?

🞎 Polystyrene, phenolic foam

🞎 Glass or basalt wool

🞎 Natural materials (hemp, flax, straw,...)

🞎 I don't know

🞎 Other:

🞎 No

1. What kind of flooring do you have in your bedroom?

🞎 Vinyl, PVC (lino)

🞎 Natural linoleum and marmoleum

🞎 Parquet, wood

🞎 Tiles

🞎 Cork, laminate

🞎 Carpet

🞎 I don't know

🞎 Other:

1. How do you heat the living rooms? (You can select more than one source)

🞎 Gas heating

🞎 Electric heating, storage heaters, direct-fired heaters

🞎 Coal or coke heating

🞎 Heating with wood, biomass

🞎 Central district heating

🞎 I don't know

🞎 Other:

1. Is this heating source (boiler, stove, etc.) located in the occupied part of the house?

🞎 Yes

🞎 No

1. Do you use air conditioning in the dwelling?

🞎 Yes

🞎 No

1. Do you use a humidifier in your apartment?

🞎 Yes

🞎 No

1. In the last 12 months, has there been visible mold (in an area larger than a credit card), moisture, or a musty odor in the living quarters?

🞎 Yes, mould

🞎 Yes, dampness

🞎 Yes, mildew odor

🞎 No

1. How often do you have windows open in your home?

In summer: 🞎 Very often open 🞎 Sometimes open 🞎 Mostly closed

In winter: 🞎 Very often open 🞎 Sometimes open 🞎 Mostly closed

1. How often do you vacuum your home?

🞎 Daily

🞎 Several times a week

🞎 Several times a month

🞎 Several times a year

🞎 Not at all

1. How often do you mop the floors?

🞎 Daily

🞎 Several times a week

🞎 Several times a month

🞎 Several times a year

🞎 Not at all

1. How often do you dust?

🞎 Daily

🞎 Several times a week

🞎 Several times a month

🞎 Several times a year

🞎 Not at all

1. How often do you use carpet cleaners?

🞎 Daily

🞎 Several times a week

🞎 Several times a month

🞎 Several times a year

🞎 Not at all

1. How often do you use floor treatment products (wax, balm)?

🞎 Daily

🞎 Several times a week

🞎 Several times a month

🞎 Several times a year

🞎 Not at all

1. Do you smoke?

🞎 Yes → If yes:

Do you smoke: 🞎 1-5 cigarettes per day 🞎 More than 5 cigarettes per day 🞎 Occasionally Do you smoke: 🞎 At home 🞎 On the balcony 🞎 Outside

🞎 No

1. Have you smoked regularly in the past?

🞎 Yes → If yes, how long ago did you stop?

🞎 Less than a week ago

🞎 Less than a month ago

🞎 Less than a year ago

🞎 More than a year ago

🞎 No

1. What is the age of the persons living in your household?

| Number of persons | Age |
| --- | --- |
|  | 0-12 years |
|  | 13-18 years |
|  | 19-25 years |
|  | 26-40 years |
|  | 41-60 years |
|  | 60 and over |

1. Do you have a pet?

🞎 Yes → If yes, which one?

| **Pet** | **Number** | **Does the pet stay in your household?** | **Does it sleep in your bed with you?** |
| --- | --- | --- | --- |
|  |  | Yes / No | Yes / No |
|  |  | Yes / No | Yes / No |
|  |  | Yes / No | Yes / No |
|  |  | Yes / No | Yes / No |
|  |  | Yes / No | Yes / No |
|  |  | Yes / No | Yes / No |
|  |  | Yes / No | Yes / No |

🞎 No

1. Have you been diagnosed with any autoimmune disease?

🞎 Yes → If yes, which one:

🞎 No

1. Have you been diagnosed with any chronic or serious illness?

🞎 Yes → If yes, which one:

🞎 No

1. Do you suffer from any of the following allergies?

🞎 Dust

🞎 Pollen

🞎 Animals

🞎 No

1. Do you suffer from any of the listed diseases?

🞎 Asthma

🞎 Chronic rhinosinusitis

🞎 Allergic rhinosinusitis

🞎 Allergic rhinitis

🞎 Nasal polyps and cysts

🞎 Cystic fibrosis

🞎 Chronic obstructive pulmonary disease (COPD)

🞎 Atopic eczema

🞎 Tuberculosis

🞎 Lung cancer

1. Do you have a cold on the day of sampling?

🞎 Yes

🞎 No

1. Do you have breathing problems on the day of sampling?

🞎 Yes

🞎 No

1. Do you breathe through your mouth during sleep?

🞎 Yes

🞎 No

🞎 I don't know

**Thank you for participating in the study.**
