# Supplementary material for: Enrichment of human nasopharyngeal bacteriome with bacteria from dust after short-term exposure to indoor environment: a pilot study
Source: BMC Microbiol. 2023 Jul 31;23:202. doi: 10.1186/s12866-023-02951-5 (PMC10391871; doi:10.1186/s12866-023-02951-5)
Supplement: Supplementary file 9 — Additional file 9. Sample rarefaction curves plotted on both unfiltered and filtered data using rarecurve function of R package vegan v. 2.6.4. [file 12866_2023_2951_MOESM9_ESM.pdf]

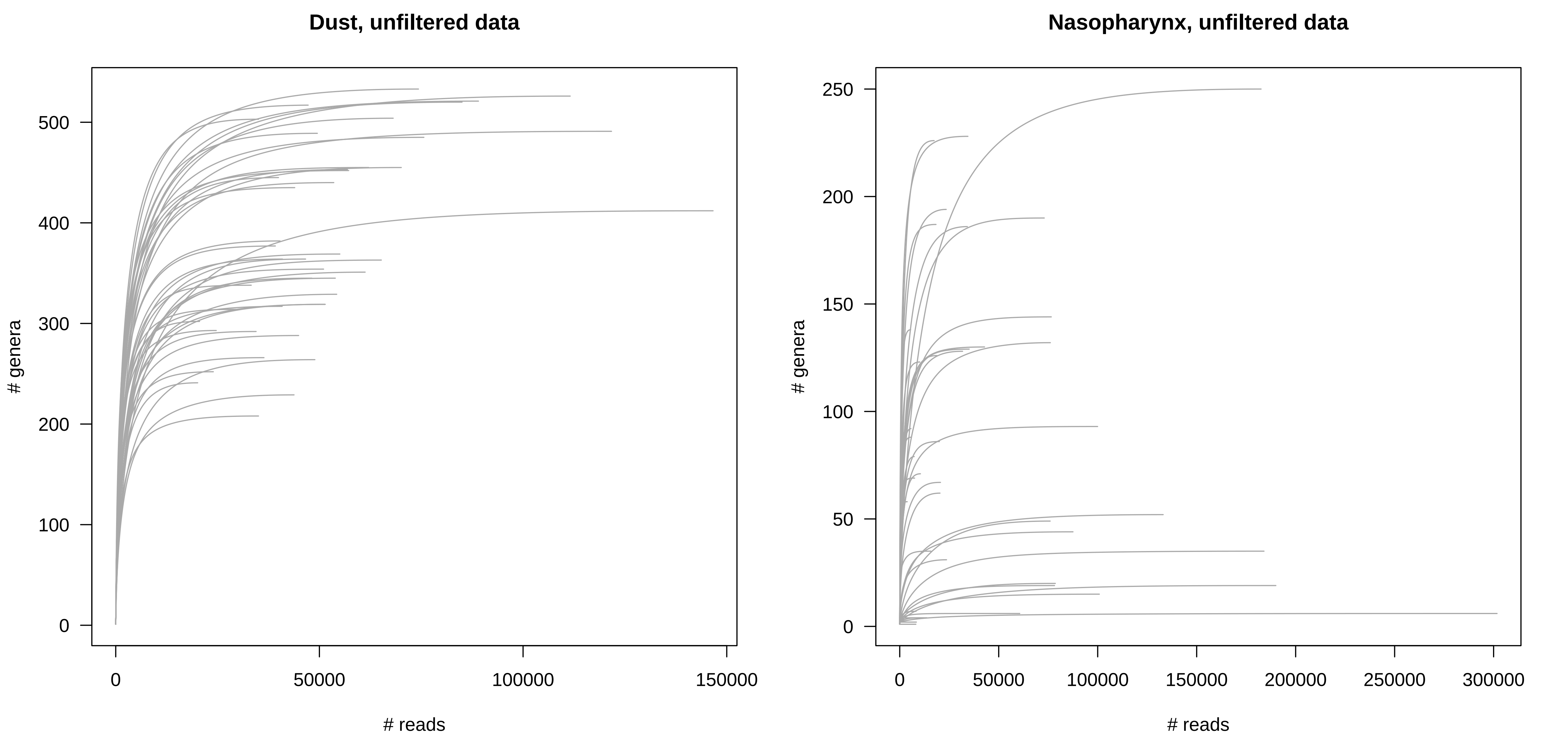

Dust, unfiltered data
genera
reads
0
100
200
300
400
500
0
50000
100000
150000
Nasopharynx, unfiltered data
genera
reads
0
50
100
150
200
250
0
50000
100000
150000
200000
250000
300000

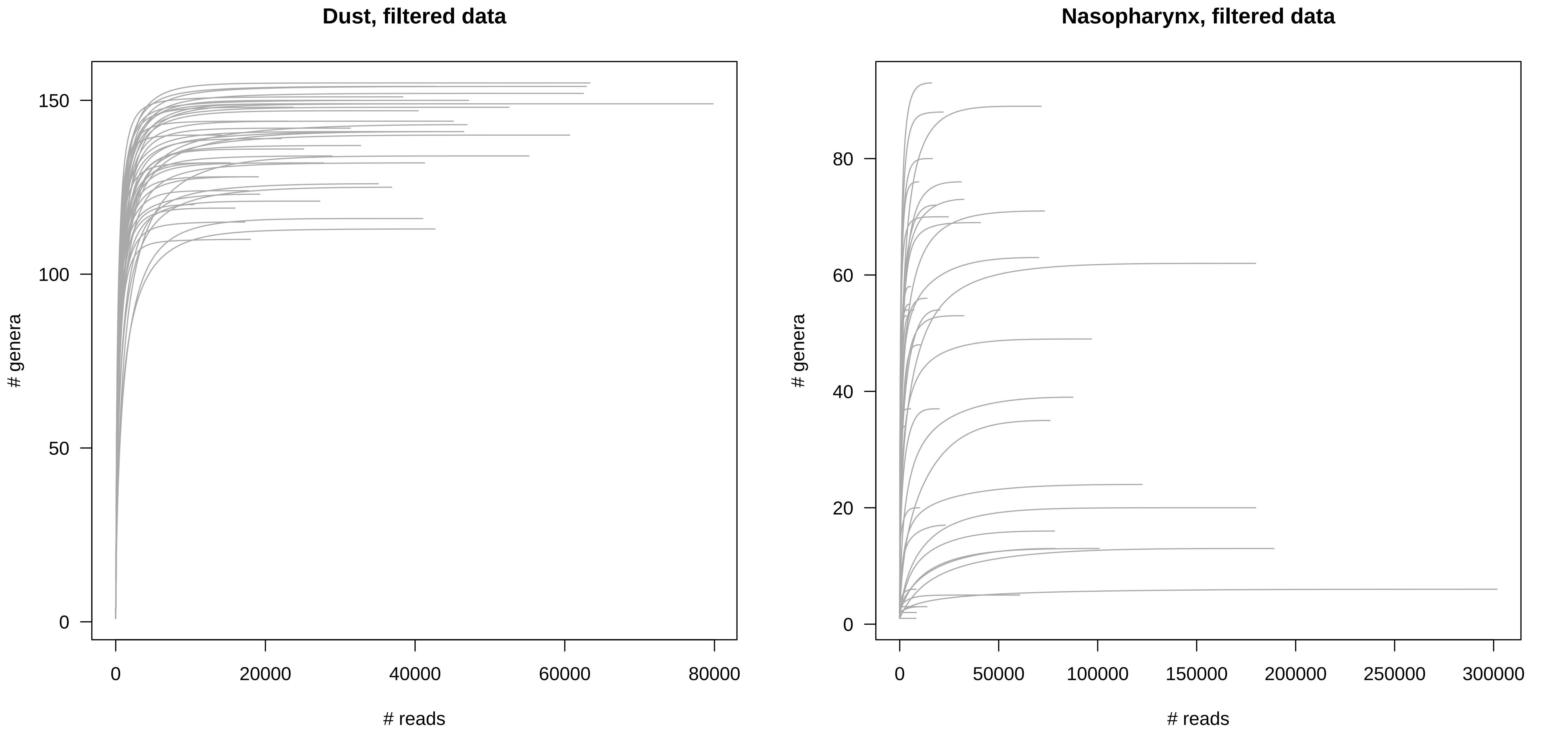

Dust, filtered data
genera
reads
0
50
100
150
0
20000
40000
60000
80000
Nasopharynx, filtered data
genera
reads
0
20
40
60
80
0
50000
100000
150000
200000
250000
300000
